# Whole exome sequencing diagnosing syndromic and non-syndromic hearing loss with expansion of the phenotypic spectrum related to *TMC1* variants

**DOI:** 10.1007/s00431-025-06052-5

**Published:** 2025-03-18

**Authors:** Nagham M. Elbagoury, Engy A. Ashaat, Mona K. Mekkawy, Ragaey Y. Mohamed, Anas M. Askoura, Peter M. Milad, Mona L. Essawi

**Affiliations:** 1 Department of Medical Molecular Genetics, National Research Centre, Giza, 12311 Egypt; 2 Department of Clinical Genetics, National R esearch Centre, Giza, Egypt; 3 Department of Human Cytogenetics, National Research Center, Giza, Egypt; 4 Department of Otorhinolaryngology, Faculty of Medicine, Beni-Suef University, Beni-Suef, Egypt; 5 Department of Otorhinolaryngology, Faculty of Medicine, Ain Shams University, Cairo, Egypt

**Keywords:** *ILDR1*, *MYO3A*, *KCNQ1*, *PEX6*, *GJB2*, SNHL

## Abstract

Hearing loss (HL) is an impending disorder. The high incidence of congenital genetic HL affects the language and communication skills of a large number of children worldwide. Our study is mainly concerned with exploring the genetic etiology of congenital hearing loss through Sanger sequencing of the coding exon in *GJB2*, the most common causative gene worldwide, in 17 patients from 13 unrelated families followed by whole exome sequencing for cases showing biallelic wildtype *GJB2*. Eleven patients from eight families showed homozygous and compound heterozygous variants in the *GJB2* gene. Six patients from five families proceeded to whole exome sequencing. One of them showed a reported variant in *ILDR1*, and three showed novel variants in the *TMC1* and *KCNQ1* genes*.* Two showed variants reported for the first time in HL patients in the *PEX6* and *MYO3A* genes.

In* conclusion*, this study suggests new insights into the contribution of *MYO3A*, *KCNQ1*, and *PEX6* to congenital sensorineural hearing loss as well as possible expansion of the phenotypic spectrum of the *TMC1* gene.

**Table:** 

**What is Known:** • *Sanger sequencing and whole exome sequencing are used for molecular diagnosis of syndromic and non-syndromic types of hearing loss (HL).* • *TMC1 gene causes a type of non-syndromic HL*.	
**What is New:** • *Expanding the molecular spectrum of MYO3A, PEX6, TMC1, and KCNQ1 genes as contributor genes in HL by detecting variants first time to be detected in HL patients.* • *Expanding the clinical spectrum of TMC1 gene to cause syndromic and non-syndromic HL.*

**What is Known:**

• *Sanger sequencing and whole exome sequencing are used for molecular diagnosis of syndromic and non-syndromic types of hearing loss (HL).*

• *TMC1 gene causes a type of non-syndromic HL*.

**What is New:**

• *Expanding the molecular spectrum of MYO3A, PEX6, TMC1, and KCNQ1 genes as contributor genes in HL by detecting variants first time to be detected in HL patients.*

• *Expanding the clinical spectrum of TMC1 gene to cause syndromic and non-syndromic HL.*

## Introduction

Hearing loss (HL) is the most common sensory disorder in humans [1–3]. It can be classified according to onset into congenital, prelingual, and postlingual. Prelingual form has a relatively high incidence of one in 500 [4]. Hearing in infants is critical for language development and communication skills. Congenital HL has an incidence of 1.5 per 1000 newborns [5]. HL can be classified according to anatomical site, severity, frequency, progression, and symmetry. Another classification is based on deafness being acquired or genetic where the genetic type represents more than 50% of HL cases. Genetic HL can be further subclassified into syndromic and non-syndromic forms where syndromic cases exceed 400 syndromes [6]. Autosomal recessive non-syndromic hearing loss (ARNSHL) is the most common form of non-syndromic HL comprising more than 100 genes (https://hereditaryhearingloss.org/recessive).

The *GJB2* gene is considered the most common causative gene among many populations [7]. Taking this background into consideration, we decided to carry out clinical and molecular delineation for a cohort of SNHL patients first by the Sanger sequencing of the *GJB2* gene followed by whole exome sequencing (WES) for negative cases in an attempt to reach a more precise diagnosis for Egyptian SNHL patients. Diagnosis of the patients on a molecular level not only benefits the patients but also helps their families through genetic counseling, carrier detection, and premarital and prenatal counseling. Early diagnosis of affected neonates can help in early therapeutic interventions such as cochlear implantation to avoid any delay in speech or any other cognitive milestones.

## Patients and methods

Seventeen patients from 13 unrelated families have been recruited from hearing loss cases referred to the otorhinolaryngology clinic at the Faculty of Medicine, Beni-Suef and Ain Shams Universities as well as the Clinical Genetics clinic at the National Research Centre (NRC) from January 2022 to December 2023. Written informed consent was signed by the parents according to the Declaration of Helsinki. The study was approved by the Medical Research Ethical Committee of Beni-Suef University (FMBSUREC/01102024/Youssef).

### Clinical evaluation

All patients went through thorough clinical examination, history taking to exclude environmental causes for HL, and three-generation family pedigree construction, as well as auditory brain stem response (ABR) to detect the severity of the hearing loss.

### Conventional cytogenetic analysis

Conventional cytogenetic analysis of blood lymphocytes was performed for P2 and P3 from the same family who had dysmorphic features to exclude chromosomal abnormalities, using the standard GTG banding technique [8]. Karyotypes were captured using an Axioskop Zeiss microscope. A total of 20 well-banded metaphase plates were analyzed and karyotyped according to the International System for Human Cytogenenomic Nomenclature recommendations [9].

### Molecular analysis

#### Sanger sequencing for *GJB2* gene

Genomic DNA (gDNA) was extracted from peripheral blood leukocytes using the salting out protocol [10]. A primer pair was designed using primer3 (https://primer3.ut.ee/) to amplify the coding exon of the *GJB2* gene and the flanking intronic sequence using the GenBank sequence (accession number: NM_004004.6) forward primer: GCTTACCCAGACTCAGAGAAG and reverse primer: CTACAGGGGTTTCAAATGGTTGC. Polymerase chain reaction (PCR) amplification followed standard procedure. Sanger sequencing followed successful PCR amplification using BigDye Terminator v3.1 Kit (Applied Biosystems, USA) on a 3500 ABI Prism DNA sequencer (Applied Biosystems). Output data was displayed by Finch TV. Sequences were aligned against the reference genome using the Basic Local Alignment Search Tool (http://blast.ncbi.nlm.nih.gov) [11].

#### Whole exome sequencing

Samples which showed wildtype sequences of the *GJB2* gene went through further molecular analysis through next-generation sequencing (NGS). The NGS technique was implemented with an output of 100 × coverage depth for > 98% of the targeted bases. The main steps include extraction of gDNA, fragmentation of the isolated nucleic acid followed by library preparation, formation of colony, sequencing, processing of data through bioinformatics tools, and finally, bioinformatics analysis of the output data. The exploration of related variants is mainly concerned with coding exons and a flanking region of 10 bases up- and down-stream in the intronic regions. Analysis of copy number variants (CNVs) was carried out using specialized software that can detect deletions and duplications spanning at least three successive exons. All potential patterns for the mode of inheritance are considered. In addition, provided clinical manifestations are used to exclude or include identified variants with respect to their pathogenicity.

## Results

**Fig. 1:**
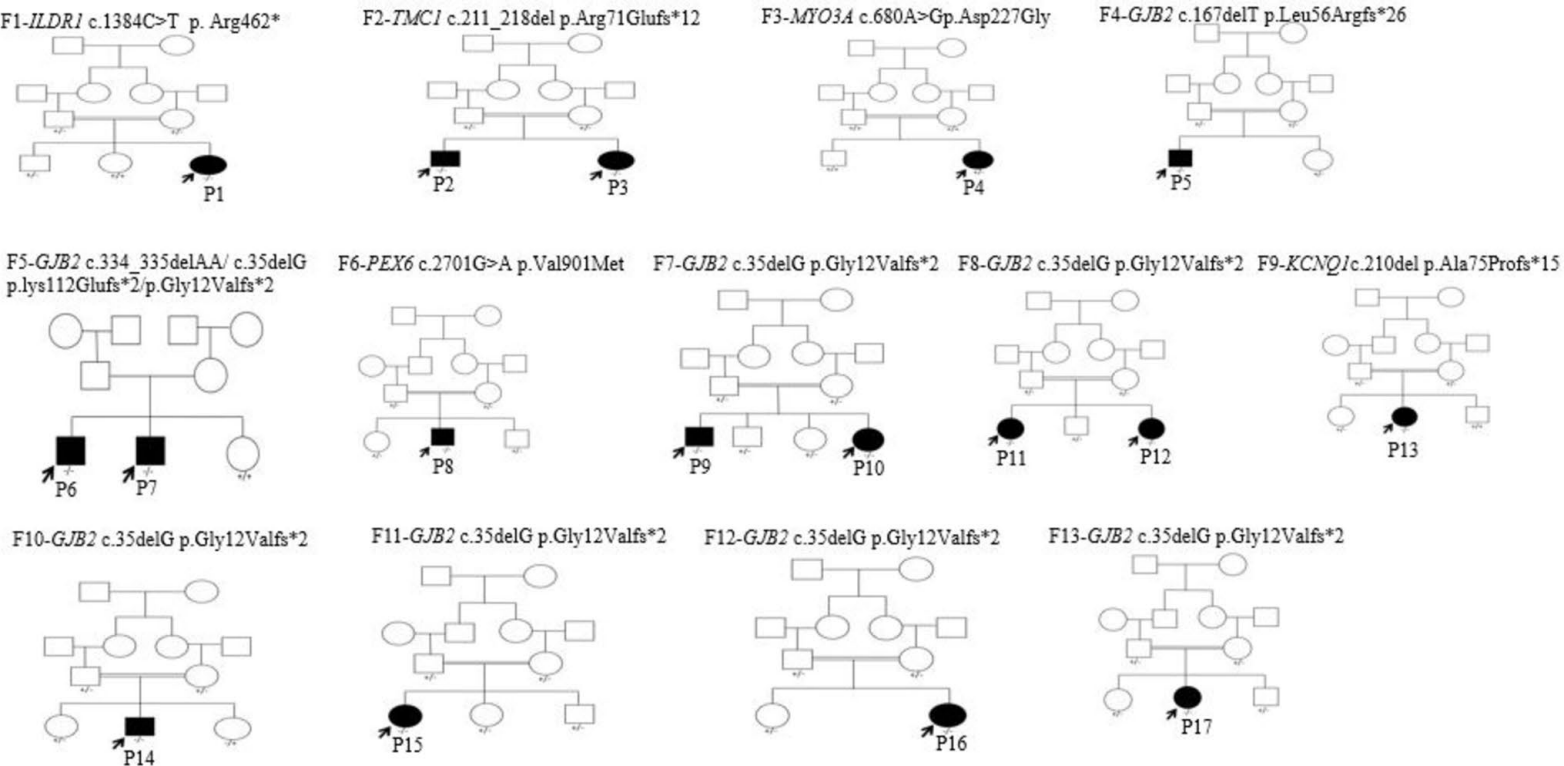
Three generations of family Pedigree for the 17 studied patients. Segregation of the variants is presented on the pedigree as + / + , + / − , and − / − (+ for normal allele and − for mutated allele). F, family; P, patient

The study included 17 patients from 13 unrelated families where females represented 47% (8/17) and males represented 53% (9/17). Consanguineous families represented 92.3% (12/13) of the studied families as shown in Fig. 1. The age of the patients ranged from 3 to 24 years at the time of referral.

**Fig. 2:**
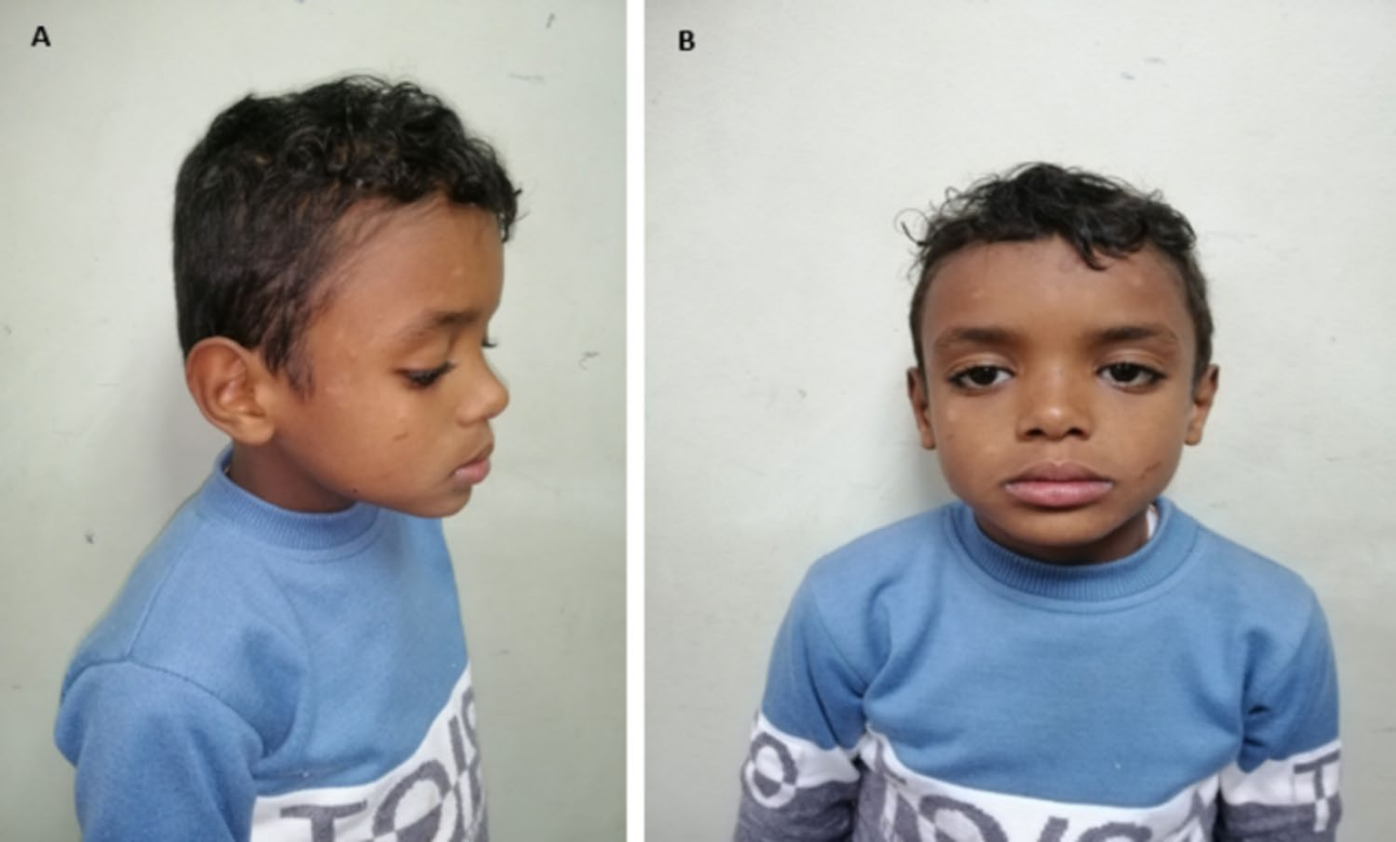
Lateral view (**A**) and frontal view (**B**) of P2 showing dysmorphic facial features including malar hypoplasia, hypertelorism, bilateral epicanthic folds, and depressed nasal bridge

All patients presented with isolated bilateral SNHL with variable severities as shown in Table 1 except for patients from families 2 and 9. P2 and P3 from family 2 presented with SNHL, dysmorphic features, mild intellectual disability (ID), and mild skin dryness (Fig. 2). Chromosomal analysis was carried out for the two patients and revealed normal results. P13 from family 9 presented with SNHL, prolonged QT interval, and syncope. All patients had non-progressive prelingual HL. All patients showed mean anthropometric measurements.

**Table 1:** Clinical and molecular results of the studied patients

Family	Patient	Gene	Amino acid change	Zygosity	Age/gender	Consanguinity	Family history	ABR	Clinical features	Phenotype (OMIM)
F1	P1	*ILDR1*	Arg462*	Homozygous	6/F	+	+	Profound HL	Non- progressive prelingual SNHL	Deafness, autosomal recessive 42 (609646)
F2	P2	*TMC1*	Arg71Glufs*12	Homozygous	7/M	+	+	Profound SNHL	Non-progressive prelingual SNHL, ID, dry skin, and dysmorphic features including bilateral epicanthic folds, hypertelorism, depressed nasal bridge, and malar hypoplasia, thick lips	Deafness, autosomal recessive 7 (600974)
	P3				13/F			Profound SNHL	Non-progressive prelingual SNHL, ID, dry skin, dysmorphic features including bilateral epicanthic folds, hypertelorism, depressed nasal bridge, and malar hypoplasia, thick lips with normal motor milestones	
F3	P4	*MYO3A*	Asp227Gly	Heterozygous	8/F	+	-	Profound SNHL	Non-progressive prelingual SNHL	Deafness, autosomal dominant 90; DFNA90 (620722)
F4	P5	*GJB2*	Leu56Argfs*26	Homozygous	5/M	+	-	Profound SNHL	Non-progressive prelingual SNHL	Deafness, autosomal recessive 1A (220290)
F5	P6	*GJB2*	lys112Glufs*2/ Gly12Valfs*2	Compound, Heterozygous	4.5/M	_	+	Profound SNHL	Non-progressive prelingual SNHL	Deafness, autosomal recessive 1A (220290)
	P7				9/M			Profound SNHL	Non-progressive prelingual SNHL	
F6	P8	*PEX6*	Val901Met	Homozygous	11/M	+	-	Profound SNHL	Non-progressive prelingual SNHL	Heimler syndrome 2 (616617)
F7	P9	*GJB2*	Gly12Valfs*2	Homozygous	14/M	+	+	Profound SNHL	Non-progressive prelingual SNHL	Deafness, autosomal recessive 1A (220290)
	P10				5/F			Rt: profound SNHL Lt: mild SNHL	Non-progressive prelingual SNHL	
F8	P11	*GJB2*	Gly12Valfs*2	Homozygous	24/F	+	+	Severe SNHL	Non-progressive prelingual SNHL	Deafness, autosomal recessive 1A (220290)
	P12				18/F			Profound SNHL	Non-progressive prelingual SNHL	
F9	P13	*KCNQ1*	Ala75Profs*15	Homozygous	3/F	+	+	Rt: moderate SNHL Lt: profound SNHL	Non-progressive prelingual SNHL, recurrent syncope, and cardiac disorder (ECG) showed prolonged QT interval	Jervel and Lange-Nielsonsyndrome1 (220400)
F10	P14	*GJB2*	Gly12Valfs*2	Homozygous	7/M	+	+	Bilateral severe to profound SNHL	Non-progressive prelingual SNHL	Deafness, autosomal recessive 1A (220290)
F11	P15	*GJB2*	Gly12Valfs*2	Homozygous	3/F	+	+	Bilateral severe to profound SNHL	Non-progressive prelingual SNHL	Deafness, autosomal recessive 1A (220290)
F12	P16	*GJB2*	Gly12Valfs*2	Homozygous	7/M	+	+	Bilateral severe to profound SNHL	Non-progressive prelingual SNHL	Deafness, autosomal recessive 1A (220290)
F13	P17	*GJB2*	Gly12Valfs*2	Homozygous	8/F	+	+	Bilateral severe to profound SNHL	Non-progressive prelingual SNHL	Deafness, autosomal recessive 1A (220290)

*F* family, *P* patient, *Rt* right, *Lt* left, *OMIM* online Mendelian inheritance in man, *M* male, *F* female, *SNHL* sensorineural neural hearing loss, *ECG* electrocardiogram, *ID* intellectual disability

**Fig. 3:**
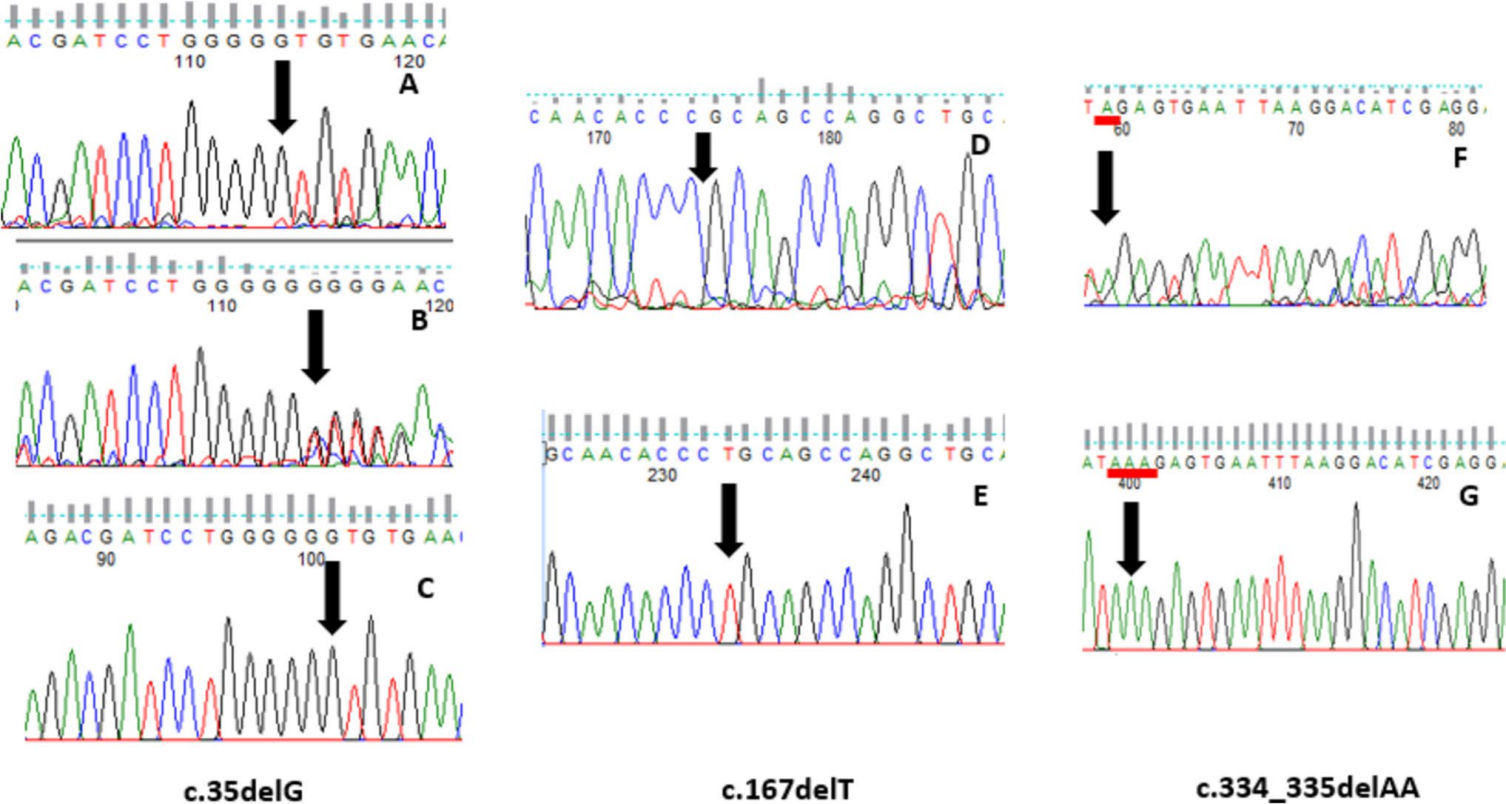
Electropherograms for the variants detected in the *GJB2* gene as follows: homozygous c.35delG (**A**), heterozygous c.35delG (**B**) against the wildtype form (**C**). Homozygous c.167delT (**D**) against the wildtype form (**E**). Homozygous c.334_335delAA (**F**) against the wildtype form (**G**)

Sanger sequencing of *GJB2* revealed the presence of previously reported pathogenic variants in 11 patients from 8 families. The most common variant (p. Gly12Valfs*2) was detected in homozygous form in eight patients from 6 families (P9, P10, P11, P12, P14, P15, P16, P17) and in compound heterozygosity with (p. lys112Glufs*2) in two patients (P6, P7) from a non-consanguineous family. One patient (P5) carried p. Leu56Argfs*26 in a homozygous form as shown in Fig. 3.

Whole exome sequencing was carried out for six patients from five families (P1, P2, P3, P4, P8 and P13). The results were conclusive for the six patients. One patient (P1) carrier a reported homozygous variant in the *ILRD1* gene (p. Arg462Ter). Three patients (P2, P3, and P13) carried novel variants where P2 and P3 carried homozygous p. R71Efs*12 variant in the *TMC1* gene. P13 carried homozygous p. Ala75Profs*15 in the *KCNQ1* gene. Two patients (P4 and P8) carried variants detected for the first time in SNHL patients. P4 carried heterozygous variant p. Asp227Gly in the *MYO3A* gene and three generations family pedigree showed no other affected family members (Fig. 1). P8 carried the homozygous p. Val901Met variant in the *PEX6* gene. In silico functional analysis for the latest four variants done using different tools supported their pathogenicity as shown in Table 2.

**Table 2:** Variants detected for the first time in SNHL patients with various prediction In silico tools supporting their pathogenicity

Variant In silico tool	NM_138691.3(*TMC1*): c.211_218del (clinvar: SCV005184325) Frameshift	NM_017433.5 (*MYO3A*): c.680A > G (clinvar: SCV005184323) Missense	NM_000287.4 (*PEX6*): c.2701G > A (clinvar: SCV005184324) Missense	NM_000218.3 (*KCNQ1*): c.210delC (clinvar: SCV005184326) Frameshift
Predicted ACMG classification (evidence)	Likely pathogenic (PVS1, PM2)	VUS (PM2, PP3)	VUS (PM2)	Likely pathogenic (PVS1, PM2)
Revel^A^	N/A	Deleterious (supporting) (0.68)	Uncertain (0.64)	N/A
MutationTaster^B^	N/A	Deleterious (1)	Deleterious (1)	N/A
DANN^C^	N/A	Deleterious (1)	Deleterious (1)	N/A
BayesDel^D^	N/A	Deleterious (supporting) (0.2)	Deleterious (supporting) (0.15)	N/A
GenoCanyon^E^	N/A	Deleterious (1)	Deleterious (1)	N/A
fitCons^F^	N/A	Deleterious (0.62)	Deleterious (0.71)	N/A
BayesDel noAF^G^	N/A	Pathogenic supporting (0.19)	Pathogenic supporting (0.15)	N/A
MetaRNN^H^	N/A	Pathogenic supporting (0.79)	N/A	N/A
EIGEN PC^I^	N/A	Pathogenic supporting (0.74)	N/A	N/A
FATHMM-MKL^J^	N/A	Pathogenic supporting (0.98)	N/A	N/A
CADD Score (V1.7)^K^	N/A	28.7	25.7	N/A
LRT^L^	N/A	Pathogenic supporting (0)	N/A	N/A
PROVEAN^M^	N/A	Pathogenic supporting (− 6.3)	N/A	N/A

^A^REVEL is an ensemble method for predicting the pathogenicity of missense variants based on a combination of scores from 13 individual tools: MutPred, FATHMM v2.3, VEST 3.0, PolyPhen-2, SIFT, PROVEAN, MutationAssessor, MutationTaster, LRT, GERP + + , SiPhy, phyloP, and phastCons. The score ranges from 0 to 1, with higher scores indicating higher liability for the variant to be disease-causing

^B^MutationTaster predicts the functional consequences of not only amino acid substitutions but also intronic and synonymous alterations, short insertion and/or deletion (indel) mutations, and variants spanning intron–exon borders

^C^DANN is a functional prediction score based on a deep neural network. The score can range from 0 to 1. Higher values are more likely to be deleterious

^D^BayesDel is a deleteriousness meta-score for coding and non-coding variants, single nucleotide variants, and small insertion/deletions

^E^GenoCanyon is a whole-genome annotation method that infers the functional potential of each position in the human genome

^F^fitCons integrates functional assays and produces a score that indicates the fraction of genomic positions demonstrating a particular pattern of functional assay results

^G^BayesDel (no AF) is a deleteriousness meta-score. The higher the score, the more likely the variant is pathogenic

^H^MetaRNN is a pathogenicity prediction score for human nonsynonymous SNVs (nsSNVs) and non-frameshift (NF) indels. It integrated information from 28 high-level annotation scores and produce an ensemble prediction model representing the likelihood of a nsSNV or NF indel being pathogenic

^I^Eigen PC: score is a function prediction score for SNVs considering allele frequencies, conservation, and deleteriousness

^J^FATHMM-MKL: Predicts noncoding effects by integrating functional annotation information from the ENCODE. Range 0 to 1

^K^Combined Annotation Dependent Depletion (CADD) scores are a tool for scoring the deleteriousness of SNVs in the human genome where higher scores indicate more deleterious variants

^L^Likelihood ratio test (LRT) predicts deleterious variants through the identification of highly conserved amino acid regions using a comparative genomics data set of 32 vertebrate species. Range 0 to 1

^M^PROVEAN: Protein Variation Effect Analyzer predicts how a variant affects the biological function of a protein. The prediction is based on alignment-based scores derived from pairwise sequence alignments between the query sequence

Through the 17 patients recruited in this study, variants were detected in six different genes (*ILDR1*, *TMC1*, *MYO3A*, *GJB2*, *PEX6*, and *KCNQ1*)*. GJB2* represented the most common causative gene through the studied patients where 64.7% of the patients (11/17 patients) carried variants in this gene. Frameshift variants were the most common variant type detected in the studied patients representing 82.3% (14/17 patients) followed by missense variants representing 11.7% (2/17 patients) and then nonsense variants representing 6% (1/17 patients) as shown in Table 3.

**Table 3:** Molecular results of all SNHL patients enrolled in the study

Family	Gene	Transcript	Nucleotide change	Protein change	Variant type	ACMG classification (criteria)	REVEL	Allele frequency (gnomAD v4.1.0)
F1	*ILDR1*	NM_001199799.2	c.1384C > T	p. Arg462*	Nonsense	Pathogenic (PVS1, PM3, PM2, PP5)	NA	**0.00002112**
F2	*TMC1*	NM_138691.3	c.211_218del	p. Arg71Glufs*12	Frameshift	Pathogenic (PVS1, PM2, PS4, PP5)	NA	No corresponding variants found
F3	*MYO3A*	NM_017433.5	c.680A > G	p. Asp227Gly	Missense	VUS (PM2, PP3)	Deleterious (Supporting) (0.68)	**0.0005111**
F4	*GJB2*	NM_004004.6	c.167delT	p. Leu56Argfs*26	Frameshift	Likely Pathogenic (PVS1, PM2, PP5)	NA	**0.0005111**
F5, F7, F8, F10, F11, F12, F13	*GJB2*	NM_004004.6	c.35delG	p. Gly12Valfs*2	Frameshift	Likely Pathogenic (PVS1, PM2, PP5)	NA	**0.007050**
F5	*GJB2*	NM_004004.6	c.334_335delAA	p. lys112Glufs*2	Frameshift	Pathogenic (PM3, PVS1, PM2, PP1, PP5)	NA	**0.000008057**
F6	*PEX6*	NM_000287.4	c.2701G > A	p. Val901Met	Missense	VUS (PM2)	Uncertain (0.64)	**0.00002664**
F9	*KCNQ1*	NM_000218.3	c.210delC	p. Ala75Profs*15	Frameshift	Pathogenic (PVS1, PM2, PS4, PP5)	NA	No corresponding variants found

*F* family, *ACMG* American College of Medical Genetics and Genomics guidelines

After a precise diagnosis of the 17 patients enrolled in the study on the clinical and molecular level, three patients (P14, P16, P17) went for cochlear implantation (CI), and follow-up of the cases proved improvement in terms of linguistic development, scholastic achievement, and verbal communication. Eight patients (P1, P4, P5, P6, P7, P8, P13, P15) will be enrolled in the national program of CI where the patients undergo the necessary investigations including high-resolution computed tomography (CT) temporal bone, cochlear implant magnetic resonance imaging (MRI) protocol, and intelligence quotient (IQ) testing. The remaining six patients (P2, P3, P9, P10, P11, P12) refused surgical intervention preferring to use sign language.

## Discussion

HL is a hampering disorder. It can be caused by environmental factors or due to genetic causes. Genetic HL represents more than 50% of the HL cases. The majority of these are non-syndromic cases where HL is not associated with any other clinical manifestation. More than 160 genes are indulged in the genetic HL pathophysiology. The *GJB2* gene is considered the most common causative gene for ARNSHL worldwide. It is highly expressed in the cochlea in the inner ear. The *GJB2* gene encodes connexin 26 protein which is one of the proteins responsible for the formation of ion channels helping in ion homeostasis in the cochlea [4, 12]. In this context, our study was conducted on a cohort of 17 patients with SNHL. Sanger sequencing was done for the *GJB2* gene for all patients included in our study. Among the 17 studied patients from 13 unrelated families, *GJB2* previously reported variants were revealed in 11 patients from 8 families where the most common variant p. Gly12Valfs*2 was detected in homozygous form in 8 patients from consanguineous families and in compound heterozygosity with p. lys112Glufs*2 in 2 patients from one non-consanguineous family. One patient carried p. Leu56Argfs*26 in a homozygous form. Two of the three detected variants are known to be common in other populations where p. Gly12Valfs*2 is common in the Caucasian population [13], p.Leu56Argfs*26 is common in Ashkenazi Jews [14] but p.lys112Glufs*2 is a relatively rare variant to be correlated to a certain population. These findings emphasize the predominance of *GJB2* variants as a cause of ARNSHL where the variants of this gene represented 64.7% of the detected variants in our cohort. Three of the patients carrying p. Gly12Valfs*2 variant (P14, P16, P17) went for cochlear implantation which is considered the optimum management for SNHL up till now. Follow-up showed satisfying improvement in linguistic development, scholastic achievement, and verbal communication.

The *ILRD1* gene is expressed in the inner ear’s outer and inner hair cells and has a role in hair cell’s adhesion and maintenance [4]. The *ILRD1* variant (p. Arg462*) detected in P1 was previously reported once in a Pakistani family [15]. The nonsense variant results in a truncated protein 84 amino acids shorter than the normal one. The variant was detected in only one patient in our study who showed typical ARNSHL. The patient suffered bilateral profound HL which augments the hypothesis that the variant causes complete loss of function for the produced protein which is essential for the development of the auditory hair cells and semicircular canal. *ILDR1*-dependent ARNSHL known as deafness, autosomal recessive 42 (OMIM# 609,646), is considered a rare sub-type of HL mostly reported in families of Middle-Eastern origin [16].

*TMC1* homozygous novel frameshift variant (p. R71Efs*12) was detected in P2 and P3 from the same family. The WES raw data was also checked for any CNVs or mitochondrial DNA variants of clinical relevance. Chromosomal analysis was carried out for the two patients to exclude any chromosomal abnormality on a large scale that could not be detected by WES and it showed normal karyotype. Clinical examination as well as a detailed gestation history was documented for the recruited patients and ruled out any possible environmental factors causing the clinical presentation of the patients. The detected p. R71Efs*12 variant leads to the production of truncated protein 83 amino acid (aa) long instead of the 760 aa long wildtype protein which is predicted to be *likely pathogenic* according to the American College of Medical Genetics and Genomics (ACMG) guidelines. *TMC1* is expressed in the hair cells in the cochlea and the vestibule and is important for mechanoelectrical transduction of the hair cells [17, 18]. Variants in this gene can cause deafness, autosomal dominant 36 (OMIM# 606,705), and deafness, autosomal recessive 7 (OMIM# 600,974), which are not associated with another clinical phenotype. In our patients apart from the profound bilateral SNHL, dysmorphic features including malar hypoplasia, hypertelorism, bilateral epicanthic folds, and depressed nasal bridge were observed (Fig. 2) as well as mild skin dryness and mild intellectual disability. Intellectual disability has been previously mentioned as a consequence of SNHL [19, 20] so discrimination of it being secondary to SNHL or caused by the presence of the *TMC1* gene variant has to be further investigated. Although *TMC1* contributes to hair bundle development and function in the inner ear [4], the precise function of the *TMC1* gene expressed in other organs is still not completely elucidated so justification of the phenotypic genotypic correlation in this case cannot be fully explained. However, in spite of the significant expression of *TMC1* in numerous tissues other than the inner ear such as the kidney, placenta, testis, urinary bladder, brain, and prostate [21], no genetic disorder has been attributed to the presence of pathogenic variants in this gene, other than HL. The *TMC1* gene variants were the underlying genetic cause among different ethnic groups, for example, it represents 3.4% in Pakistani patients [22, 23], 2.4% in Chinese patients [24], 3.1% in Western European patients [25], 0.5% in Dutch patients [26], 0.8% in Palestinian patients [27], 0.5% in Czech patients [28], 4.3% to 8.1% in Turkish patients [29, 30], 5.9% in Tunisian patients [31], and 0.9% in American patients [32]. In Egypt, the frequency of participation of *TMC1* variants in ARNSHL is relatively low reaching 1.6% [33].

The *KCNQ1* gene encodes a type of voltage-gated potassium channels that is highly expressed in the heart and the inner ear [34]. Pathogenic variants in the *KCNQ1* gene can lead to four autosomal dominant syndromes all of which are due to certain cardiac repolarization defects. Jervell and Lange-Nielsen syndrome (OMIM# 220,400) is the only recessive syndrome that is correlated to the gene where homozygous pathogenic variants not only affect repolarization of the heart leading to prolonged QT, syncope due to ventricular arrhythmias, and increased risk of sudden death [35] but also causes congenital deafness due to morphological anomalies in the inner ear caused by the reduction in the volume of the endolymph [34]. In our study, *KCNQ1* homozygous frameshift variant p. Ala75Profs*15 was detected in a 3-year-old girl (P13). The variant leads to a truncated protein 90 aa long instead of the 676 aa long wildtype protein. The prolonged QT interval was obvious in the ECG of the patient. On questioning the mother, she affirmed the syncopal attacks occurrence for the girl.

The *MYO3A* gene is highly expressed in the inner ear and has an important role in the development, maturation, and operation of hair cell stereocilia hair bundle–mediated mechanotransduction [4, 36]. Pathogenic variants in this gene cause deafness, autosomal dominant 90 (OMIM# 620,722), and deafness, autosomal recessive 30 (OMIM# 607,101). *MYO3A* heterozygous missense variant p. Asp227Gly was detected in one patient in our study who had isolated SNHL. The dominant mode of inheritance was proved by familial segregation. The wildtype aspartic acid is larger and less hydrophobic than the mutated glycine. The change in charge from the negatively charged aspartic acid to the neutral glycine affects a salt bridge that is normally formed with lysine at position 113 affecting the stability of the protein. On the other hand, the mutated glycine known for its high flexibility disrupts the rigidity required at this position [37]. This variant is the second dominant variant to be reported in the kinase domain and the fourth dominant variant to be reported in *MYO3A* so far [38] where the other two dominant variants are located in the motor head domain [36, 39]. It is located in 12 amino acids (aa) prior to the first reported dominant variant in the same domain p. Leu239Pro that was detected in a German family and is alleged to cause autosomal dominant HL due to a dominant-negative mechanism. The dominant mode of inheritance of p. Leu239Pro was proved by the segregation of the family members [40]. The second dominant *MYO3A* variant (p. Leu697Trp) was reported to have a non-penetrance effect in a Brazilian study that was conducted on 2 unrelated families from the southeastern region of Brazil where p. Leu697Trp dominant variant, located in the motor head domain of the *MYO3A* gene, was detected in a heterozygous form in 36 affected individuals from both families. Interestingly the variant was also detected in three unaffected family members whose ages ranged from 28 to 30 years. The study postulated a non-penetrance effect of *MYO3A* dominant variants or a compensation mechanism by the wildtype *MYO3B* gene which could exert a functional effect that counteracts the effect caused by the pathogenic variant on *MYO3A* in some individuals [39]. This compensation was also proved in mice where the loss of function of *MYO3A* is counteracted by the effect of wildtype *MYO3B* [41]. The third dominant variant reported is p. Gly488Glu, located in the motor-head domain, which was detected in four members of an African-American family. Interestingly, in contrast to our patient who presented with the common non-progressive prelingual HL, the members of this family suffered progressive post-lingual HL. The study showed that p. Gly488Glu variant reduced the ATPase activity of *MYO3A* but enhanced its motility. The progressiveness of HL in this family was justified by the accumulation of the mutant *MYO3A* which worsened the mechanotransduction dysfunction resulting in the increase of the severity of deafness by time [36].

The *PEX6* gene is one of the peroxisomal biogenesis factors encoding proteins that are vital for peroxisomal matrix and membrane proteins [42]. It is expressed in the inner ear in the outer and inner hair cells as well as auditory and vestibular ganglion neurons [43]. Pathogenic variants in the *PEX6* gene result in Heimler syndrome 2 (OMIM# 616,617), Zellweger (OMIM: 614,862), and Peroxisome biogenesis disorder 4B (OMIM# 614,863). Heimler syndrome, which is considered a rare autosomal recessive disorder, was correlated to the *PEX6* gene in 2015 [44]. It is the mildest end of the peroxisomal biogenesis disorder spectrum where patients present with sensorineural hearing loss as well as some problems in teeth and nail development. The first cases in the Middle East were reported in 2022 in Saudi Arabia where a missense variant (p.Val97Gly) was detected in two probands from two families [42]. In our patient (P8), the *PEX6* gene missense variant p. Val901Met was detected in a homozygous form. In this variant the substitution of valine with methionine at position 901 results in a less stabilized protein due to the difference between the two amino acids in size where the mutant residue has a bigger moiety leading to a reduced function protein [45]. In the case of hypomorphic variants that cause a partial loss of gene function, some clinical presentations might be very subtle [44]. In our patient, the main clinical presentation was bilateral sensorineural hearing loss with no pronounced problem in teeth or nails which can be attributed to the presence of some residual function of the mutated protein.

In conclusion, our study expands the genotypic spectrum of *MYO3A*, *KCNQ1*, *PEX6*, and *TMC1* genes. We report the probability of association of the *TMC1* gene with other phenotypic presentations other than HL suggesting expansion of its phenotypic spectrum. Functional validation is recommended to be carried out in any future study for the variants detected in *MYO3A*, *KCNQ1*, *PEX6*, and *TMC1* genes to provide stronger evidence for the pathogenicity of these variants and more robust support for their proposed role in hearing loss.

## Data Availability

The data that support the findings of this study are available from the corresponding author upon reasonable request.
